# Determination of haplotypes at structurally complex regions using emulsion haplotype fusion PCR

**DOI:** 10.1186/1471-2164-13-693

**Published:** 2012-12-11

**Authors:** Jess Tyson, John A L Armour

**Affiliations:** 1 School of Biology, University of Nottingham, Queen’s Medical Centre, Nottingham, NG7 2UH, UK

**Keywords:** CNV, *DEFA1A3*, Emulsion haplotype fusion PCR, Structural haplotype

## Abstract

**Background:**

Genotyping and massively-parallel sequencing projects result in a vast amount of diploid data that is only rarely resolved into its constituent haplotypes. It is nevertheless this phased information that is transmitted from one generation to the next and is most directly associated with biological function and the genetic causes of biological effects. Despite progress made in genome-wide sequencing and phasing algorithms and methods, problems assembling (and reconstructing linear haplotypes in) regions of repetitive DNA and structural variation remain. These dynamic and structurally complex regions are often poorly understood from a sequence point of view. Regions such as these that are highly similar in their sequence tend to be collapsed onto the genome assembly. This is turn means downstream determination of the true sequence haplotype in these regions poses a particular challenge. For structurally complex regions, a more focussed approach to assembling haplotypes may be required.

**Results:**

In order to investigate reconstruction of spatial information at structurally complex regions, we have used an emulsion haplotype fusion PCR approach to reproducibly link sequences of up to 1kb in length to allow phasing of multiple variants from neighbouring loci, using allele-specific PCR and sequencing to detect the phase. By using emulsion systems linking flanking regions to amplicons within the CNV, this led to the reconstruction of a 59kb haplotype across the *DEFA1A3* CNV in HapMap individuals.

**Conclusion:**

This study has demonstrated a novel use for emulsion haplotype fusion PCR in addressing the issue of reconstructing structural haplotypes at multiallelic copy variable regions, using the *DEFA1A*3 locus as an example.

## Background

Establishing phase and resolving combinations of variants situated on the same parental chromosome has long been considered important in human genetic studies and approaches to resolve newly sequenced diploid genomes into their haploid components are gaining momentum [1-3]. Yet, for the majority of genome sequences published to date, the fragmentation of DNA prior to sequencing, coupled with the short read length of most of the technologies, means that data is provided as a diploid, unphased read-out, thus losing intrinsic information about which parental chromosome a particular allele is located on.

The determination of individual haplotypes is valuable in many areas of research, including (i) association studies and disease risk, where the analysis of haplotypes is more powerful than the analysis of single markers alone [4] and where there may be a haplotype-dependent influence of variants on risk [5,6], (ii) evolution and population genetics [7-9] and (iii) pharmacogenomics [10]. However, despite the fundamental biological importance of sequence haplotypes, they remain relatively unexplored both on a genome-wide and a local scale, due in large part to the technical difficulties of establishing phase.

The reconstruction of maternal and paternal haplotypes over distances longer than a few kilobases is not trivial and is rarely determined unless family members are included along with the subjects [11-13]. Most commonly, haplotypes have been constructed through analysis of segregation from family data, or inferred using statistical methods. There are numerous statistical approaches for inferring phase (example algorithms highlighted in [2,14]), and whilst successful over short distances and frequently used in the post-HapMap era [9,15], these are not always reliable for regions of low linkage disequilibrium (LD) or for rare variants (2). Statistical inference of phase is particularly difficult for multiallelic copy-variable regions of the genome. Due to their dynamic nature, these regions can exhibit low LD with neighbouring SNPs due to the different haplotypic background on which any one copy number state can occur. The use of mate pair reads can permit the reconstruction of haplotype information, although often this is only partially successful [16]. Experimentally addressing phase may be approached by physically separating the two parental chromosomes prior to sequence (and statistical) analysis by construction of somatic cell hybrids [17], microdissection [18], microfluidics [19] or chromosome sorting [20]. These methods serve to provide haplotype information at the genome-wide or chromosome scale, yet they often rely on specialised instruments and expertise or are time-consuming and expensive. Other approaches to generating haplotypes on a genome-wide scale include cloning- and dilution- based methods that aim to reduce the complexity of phasing by separating the genomic DNA into pools that contain DNA that is either maternally or paternally derived, with some of the most comprehensive molecular haplotypes on a genome-wide scale produced through the use of fosmid clones [21-24]. Single-molecule sequencing and Long Fragment read (LFR) technologies could offer a definitive haplotype on a genome-wide scale, in particular those methods that offer the possibility of long read lengths and the phasing of heterozygous SNPs into long haplotype contigs [25-28]. Whilst such methods are potentially successful in determining phase of heterozygous SNPs for the majority of the genome, regions that are variable in structure cannot be simply reconstructed.

For many studies, including those at structurally complex regions of the genome, the determination of phase on a more local scale may be more useful and sufficient. These more focussed molecular approaches include SNP-specific extraction of targeted genomic regions [29], recombineering [30], and most frequently, PCR-based techniques such as long PCR, allele-specific PCR and a combination of both [31-33], Linking emulsion PCR [34,35] and Haplotype fusion and ligation haplotyping [36]. PCR-based techniques provide haplotype data on a relatively small scale, with the various methods differing in the way that the phase of variants is resolved. These methods have been valuable in determining the phase of variants, particularly in the case where haplotypes are predictive of disease risk or influence gene expression [34,36].

In emulsion haplotype fusion PCR, fusion PCR is performed to bring two regions of interest, from separate parts of the same chromosome, together into a single product. This product corresponds to a condensed haplotype with the phase of the variants maintained [35,36]. This reaction is carried out in an emulsion, which contains millions of aqueous microdroplets separated within an oil phase. Each microdroplet thus acts as an individual PCR microsystem in which amplification from single molecules of target DNA occurs independently of the other droplets, with PCR products confined to their droplet of origin. This method has previously been used to assay for chromosomal inversions [36,37] and for haplotyping SNPs [34-36]. Its value lies in the fact that the distance between the two regions or SNPs being condensed into a haplotype is theoretically only limited by the size/quality of the template genomic DNA. The independent amplification of two short amplicons, that make a fused product as the reaction occurs, circumvents issues such as decreasing efficiency and template-switching errors associated with long PCR as amplicon length increases [38]. As such, haplotype fusion PCR will allow determination of haplotypes at a much greater distance than is accurately possible by long PCR.

The emulsion haplotype fusion procedure has the potential to be used for any region of the genome to generate data when familial or computational approaches are limiting or not possible. We therefore investigated its use at structurally complex regions which are particularly hard to analyse [39]. The α-defensin locus is a particularly challenging locus at which to determine haplotypes. This locus is a multiallelic copy-variable region located on chromosome 8p23.1, with a diploid copy number range of 3–11 in Europeans [40]. The α-defensin genes, which encode important proteins in the innate immune response, are highly expressed in neutrophils and are therefore valuable in studying the relationship between gene copy number and gene expression, and the relationship between haplotype structure and expression. This structurally complex region consists of a variable number of 19kb full repeats and a 10kb centromeric “partial” repeat present in one copy per chromosome. The region on the UCSC assembly (hg18) [41] shows two copies of *DEFA1* with one copy of *DEFA3* in the most centromeric position, but this is only one of many possible structures at this locus. The observation that each gene locus within both the full and partial repeats can be occupied by either *DEFA1* or *DEFA3* has led to the designation of the locus as *DEFA1A3*[40]. In addition to this, sequence variants within the genes themselves increase the complexity of the locus and further complicate the reconstruction of a structural haplotype. As for many copy number variants (CNVs), whilst diploid copy number can be determined relatively straightforwardly [42,43], positional information regarding the order of genes, and the sequence variants contained within them, with the phase of SNPs either side of the CNV is often overlooked. To address this issue, we have developed an emulsion haplotype fusion PCR approach for linking two PCR products each of up to 1kb in length and examined its use to reconstruct structural haplotypes at the copy-variable locus *DEFA1A3*.

## Methods

### DNA

DNA samples used to determine the structural haplotypes at the *DEFA1A3* locus were HapMap CEU NA06990, NA12760 and NA12878. All HapMap samples were obtained from the Coriell Institute for Medical Research (http://ccr.coriell.org/).

### Haplotype-fusion PCR

**Figure 1:**
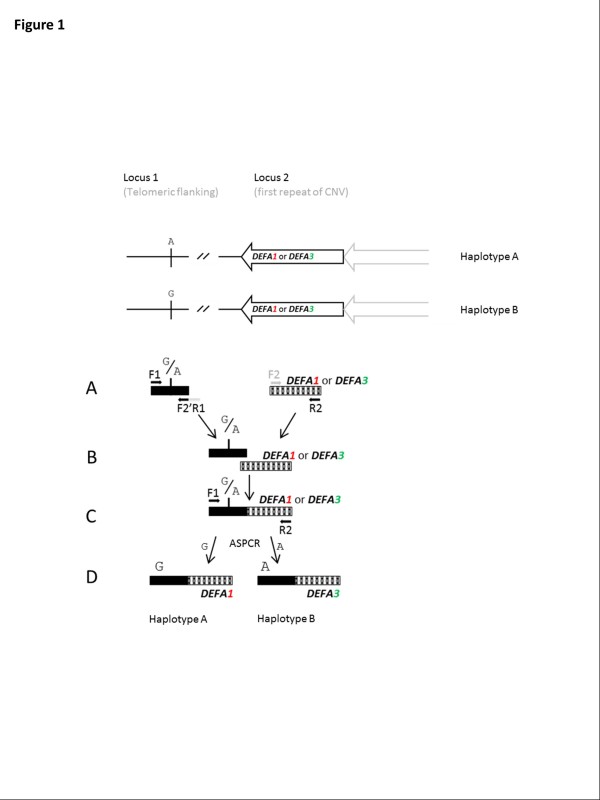
**Emulsion haplotype fusion PCR.** Two loci, approximately 12kb apart are illustrated. Locus 1 corresponds to a single-copy region flanking the copy number variable region containing *DEFA1A3* (locus 2). *DEFA1A3* repeat units are illustrated as arrows, with a black arrow representing the most telomeric repeat, and a grey half arrow representing another repeat unit present to the right. Amplicons to be fused are shown in A-D: **A.** Independent amplification occurs at locus 1 and locus 2, with primers F1, F2’R1 and R2 used in the emulsion. Primer F2 is shown in light grey, and is not used in the emulsion but is necessary for the design of the tailed primer **B.** Locus 1 amplicon primes on locus 2, creating the fusion product. **C.** Fused product is amplified with F1 and R2. **D.** Allele-specific PCR (ASPCR) with nested primers allows determination of each haplotype by sequencing.

Figure 1 illustrates the principle of haplotype fusion PCR using the telomeric end of the *DEFA1A3* CNV as an example. Two pairs of primers are designed to independently amplify regions containing informative SNPs (locus 1) and sequence variants within the copy-variable gene (locus 2). These primers are denoted F1 and R1 (locus 1 amplicon) and F2 and R2 for the locus 2 amplicon. The sequence corresponding to the reverse complement of the forward primer from locus 2 (F2) is appended to the reverse primer from locus 1 (R1) to make a tailed primer, F2’R1 [44]. Primers F1, F2’R1 and R2 are the primers used in the emulsion PCR stage, with F2’R1 included at a low concentration. In the reaction, exponential amplification occurs at locus 1 (between primers F1 and F2’R1) with linear amplification from R2 (locus 2) (Figure 1A). The presence of a tail on primer F2’R1, which is complementary to F2 (locus 2), results in a product with the sequence of F2 at the 3’ end. This enables the top strand of the locus 1 amplicon to prime the locus 2 amplicon (Figure 1B), thus resulting in the formation of a fused product (Figure 1C). Using F2’R1 at low concentration ensures this primer becomes exhausted, such that successive PCR cycles generate copies of the fused product using primers F1 and R2 which are in the reaction at a higher concentration. Carrying out the reaction in an emulsion ensures that fusion only occurs between products originating from the same starting molecule, i.e., from the same haplotype. Allele-specific PCR (ASPCR) with nested primers allows determination of haplotype by sequencing (Figure 1D).

### Primer design

**Figure 2:**
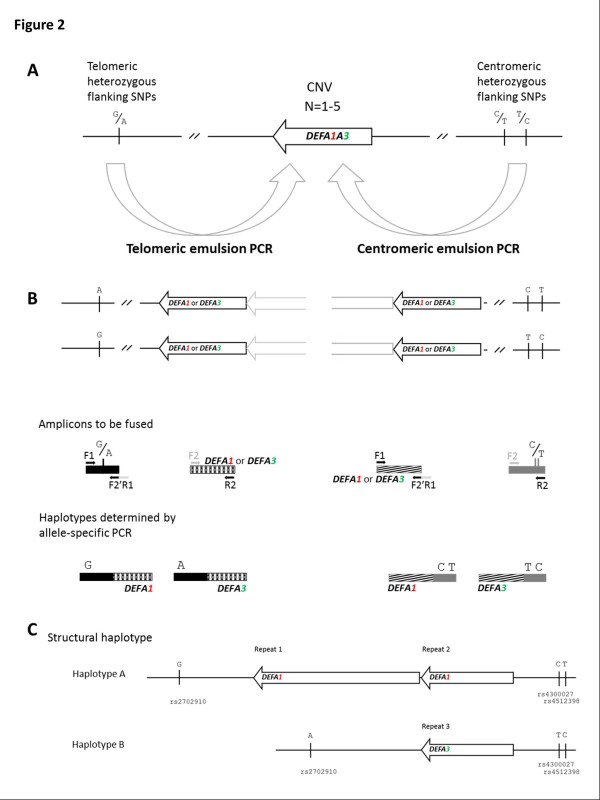
**Emulsion haplotype fusion PCR for determination of structural haplotypes.** Two emulsion haplotype fusion systems are used to provide spatial information either side of the CNV as shown by the two large arrows (**A**). Amplicons are designed to fuse non copy-variable flanking regions to the gene in the first repeat, from either the telomeric or centromeric directions independently (**B**). *DEFA1A3* repeat units are illustrated as arrows, with a black arrow representing the most telomeric or centromeric repeat, and a grey half arrow representing another repeat unit present to the right (for the telomeric system) or left (for the centromeric system). Amplicons to be fused and the final haplotypes are shown as filled rectangles. Primers used in the emulsion are shown. Integration of the telomeric and centromeric sequence data is used to determine the structural haplotype across the CNV (**C**). This is shown for NA06990.

Emulsion haplotype fusion systems were designed to link flanking regions to the first gene in the centromeric (shown on the right) and telomeric (shown on the left) repeat positions of the CNV region. Figure 2A illustrates the concept of designing fusion systems within 2-copy regions/non-copy variable regions either side of the CNV to fuse to amplicons within the CNV and thus provide a structural haplotype for either end of the CNV.

#### Centromeric emulsion PCR

Information on the centromeric flanking region and associated informative SNPs was obtained by sequencing (Holly Black, personal communication). For linking these SNPs to the gene in the most centromeric repeat (Figure 2B), individual primers were designed to amplify a 582bp amplicon from *DEFA1A3* and a 227bp product encompassing rs4300027 (Chr8:6867985) and rs4512398 (Chr8:6868004) (UCSC genome browser, March 2006 [41]). *DEFA1A3* sequence variants were identified by PCR and sequencing of the 582bp amplicon (see Additional file 1: Table S1 for details and location of *DEFA1A3* sequence variants).

#### Telomeric emulsion PCR

Information on telomeric flanking SNPs was obtained by downloading phased haplotype data (HapMap, release #24) for a 10.7kb region (chr8:6801434–6812149, UCSC genome browser, March 2006 [41]) telomeric to the start of the first full repeat. Within this 10.7kb region, two different PCR amplicons were designed to incorporate at least 2 informative variants in the HapMap CEPH individuals used in this study. For NA12760 and NA06990, telomeric emulsion system 1 was used to establish haplotypes for the telomeric end of the copy-variable region (Figure 2B). A 771bp PCR product was designed to include SNPs rs2738046 (Chr8:6810952), rs2738045 (Chr8:6810978) and rs2702910 (Chr8:6811301) telomeric to the copy variable region. For NA12878, telomeric emulsion system 2 was used to establish telomeric haplotypes. A 376bp PCR product designed to include SNPs rs56342413 (Chr8:6808918) and rs2738058 (Chr8:6809027), heterozygous in NA12878, telomeric to the copy variable region. Both products were fused to a 582bp amplicon from *DEFA1A3*.

For the second round of amplification, once the emulsion has been disrupted, both nested and allele-specific primers were designed to amplify and characterise the fused products generated in the emulsion (see Additional file 1: Table S2 for details). Combining data from both emulsion systems allows a structural haplotype to be assembled (Figure 2C).

All primers were manufactured by Invitrogen or IDT with the desalt purification option (See Table 1 for primer sequences).

**Table 1:** Primers used in the first round of emulsion haplotype fusion PCR

	**Primer name**	**Primer sequence (5’-3’)**
Centromeric Emulsion PCR	F1	AATGCACGCTGGTATTCTGCAA
	F2’R1	TTCTCTAGCCCATCCTTGCAGGCGGGAGAGAGGTTCCAGAGTTG
	R2	TGGTGTTGGCTCAGCTGGAA
Telomeric Emulsion PCR system 1	F1	TTCATTGGCCACCCTGGACT
	F2’R1	TTGCAGAATACCAGCGTGCATTCCCCCTGGAAACCTCAATCC
	R2	CGGGAGAGAGGTTCCAGAGTTG
Telomeric Emulsion PCR system 2	F1	TGCCTCAGTCTTCCCCAAAG
	F2’R1	TTGCAGAATACCAGCCTGCATTTTACCAGACGCCACCAGTCA
	R2	CGGGAGAGAGGTTCCAGAGTTG

### PCR

For all emulsion experiments, PCR conditions were established in solution prior to being carried out in an emulsion. PCRs were prepared in a total aqueous volume of 100 μl, containing 1x Phusion GC buffer (Finnzymes), 200 μM each dNTP (NEB), 1 μM primer F1, 1 μM primer R2, 25 nM primer F2’R1, 200 ng genomic DNA and 14 units Phusion DNA polymerase (Finnzymes). 10 μl aqueous phase was added every 5 to 10 seconds to 200 μl oil phase (4.5% vol/vol Span 80, 0.4% vol/vol Tween 80 and 0.05% Triton X-100 dissolved in light mineral oil (Sigma)) in a 2 ml cryo-vial while stirring at 1000 rpm for a total of 5 minutes as previously described by Turner *et al.*[44]. The average diameter of the aqueous droplets was about 5 μm, as ascertained by microscopy (data not shown). 100 μl of aqueous/oil mixture was transferred to 0.5 ml PCR tubes and first-round amplification carried out at 98°C for 30 seconds and 40 cycles of 98°C for 10 seconds, 70°C for 30 seconds and 72°C for 30 seconds. This was followed by 72°C for 5 minutes and a 4°C hold. 100 μl of the original aqueous/oil mixture was not subjected to first-round thermal cycling, and retained as a non-cycle emulsion control at the second stage of PCR. Emulsions were disrupted as soon as possible after the final cycle of the PCR by adding 200 μl hexane (Sigma) directly to the PCR tube, vortexing for 20 seconds and centrifuging at 13,000 g for 3 minutes. After removal of the oil phase, the hexane extraction was repeated twice more. The non-cycle emulsion control, i.e. 100 μl of the original aqueous/oil mixture that had not undergone the first PCR, was extracted alongside the emulsion PCRs and used in the secondary PCR as a control. Before proceeding to the secondary PCR (or storing at −20°C), uncovered tubes were left for 10 minutes in the fume hood to allow any remaining hexane to evaporate.

### Allele-specific PCR (ASPCR) and sequencing of condensed haplotypes

After hexane extraction, the primary (fusion) products in the remaining aqueous phase were diluted 10-fold with dH_2_O for the second round of amplification. At this stage extracted aqueous phase from the non-cycle emulsion and 10 ng of genomic DNA were included as two additional controls. A lack of amplification derived from these controls demonstrates that the product is dependent on both rounds of PCR and that the cycling conditions used do not permit the formation of the fused product in the absence of these steps. 1 μl of the 1:10 dilution was amplified in a total volume of 20 μl containing 1x NH_4_ reaction buffer (Bioline), 2 mM MgCl_2_, 200 μM each dNTP, 500 nM of each primer F1 or F1N and R2N and 1 unit of Taq DNA polymerase (Bioline). Amplification of this secondary product was carried out for 30 cycles of 95°C for 30 seconds, primer-specific annealing step 1 minute and 72°C for 1 minute. Annealing temperatures and primers used in the second round of PCR are detailed in Additional file 1: Table S2. PCR conditions for allele-specific primers were established using control DNA samples homozygous for each genotype.

PCR products were purified using AMPure (Agencourt) following the manufacturer’s protocol and 20-30 ng sequenced, in both the forward and reverse direction, using 0.5 μl BigDye Terminator v3.1 mix and 3.5 μl 5x BigDye sequencing buffer (Applied Biosystems) with standard cycle sequencing conditions. Extension products were purified using CleanSEQ (Agencourt) following the manufacturer’s protocol.

## Results and discussion

The lengths of the amplicons attempted in previously published emulsion fusion experiments have been relatively short, and in turn have limited the amount of SNP information that can be assembled into haplotypes [35,36]. To be able to use emulsion haplotype fusion PCR to establish structural haplotypes, it was necessary both to increase the length of the amplicons fused in the emulsion (to allow the phasing of more SNPs simultaneously) and to simplify post-fusion detection of the phase. Conditions were established such that amplicons of up to 1kb in length, (and hence a 2kb fused haplotype) could be reliably and reproducibly achieved. In contrast to previously published methods of emulsion fusion PCR [34,36], PCR and sequencing were introduced to detect the resulting phase. Once established in a test system, the aim was to investigate whether this method could be used to reconstruct structural haplotypes at the multiallelic copy-variable locus, *DEFA1A3*.

Using the *CCL3L1*/*CCL4L1* locus as a test system, preliminary work explored aspects of the emulsion haplotype fusion PCR that were important for its reliability and reproducibility (see Additional files 1 and 2). The effects of the average droplet size of the aqueous compartment in the emulsion and concentration of primers in the reaction were explored, in particular the concentration of the fusion primer F2’R1, where 25 nM gave the best results. Amplification of the fused product was only observed after two rounds of PCR; the first round, which is carried out in the emulsion, and a second, once the emulsion has been disrupted. It is at this second stage that the use of at least one nested primer ensured a specific secondary product. In our experience, a predominance of unwanted products of incorrect size is observed if the same pair of primers is used in both the emulsion PCR stage and the secondary amplification. An additional control was introduced, which consisted of retaining a 100 μl aliquot of each emulsion that was not amplified in the first round of PCR. The aqueous phase for this control was extracted as for the emulsion PCR product, and resultant aqueous phase amplified only in the secondary PCR. This resulted in the absence of a PCR product thus demonstrating that amplification depended on the emulsion PCR stage.

Having demonstrated the successful fusion of two 1kb amplicons in an emulsion, and direct sequencing of the product to confirm the maintenance of phase using the known relationship of SNPs in the *CCL3L1*/*CCL4L1* test system (see Additional files 1 and 2), the method was applied to the *DEFA1A3* locus. As is the case for many multiallelic copy-variable regions, multiple levels of complexity exist at the *DEFA1A3* region in the form of copy number variation, gene identity and sequence variation [40]. We wanted to establish whether emulsion haplotype fusion PCR could be used to reconstruct haplotypes and provide positional information about the location of the genes and associated sequence variants across this copy-variable region. For the samples studied, diploid copy number and number of *DEFA1* and *DEFA3* genes was determined by paralogue ratio test (PRT) (manuscript in preparation) and allele-ratio analysis respectively [40].

Initial work centred on a 3-copy individual, NA06990, for which haplotype copy number and ratio of *DEFA1*:*DEFA3* genes had previously been established through segregation (Figure 2C; Fayeza Khan, personal communication). Therefore, prior to this study, it was known that NA06990 has a 2-copy haplotype consisting of 2 copies of *DEFA1*, and a 1-copy haplotype with a single copy of *DEFA3* occupying the centromeric partial repeat location. This sample served as a test of whether emulsion haplotype fusion PCR could reconstruct linear haplotypes faithfully.

In order to build up the structural haplotype, sequence variants within the gene were first identified by (non-emulsion) PCR and sequencing using the centromeric emulsion primers F1 to F2’R1. Sequence variants and browser coordinates are shown in Additional file 1: Table S1. This provided information on the number and nature of sequence variants present in the three copies of *DEFA1A3* in this individual, but not which combinations of variants were found in the same gene or where each gene was located. Heterozygosity for flanking SNPs was determined by PCR and restriction enzyme digest or by using HapMap phased genotype data (release #24).

**Figure 3:**
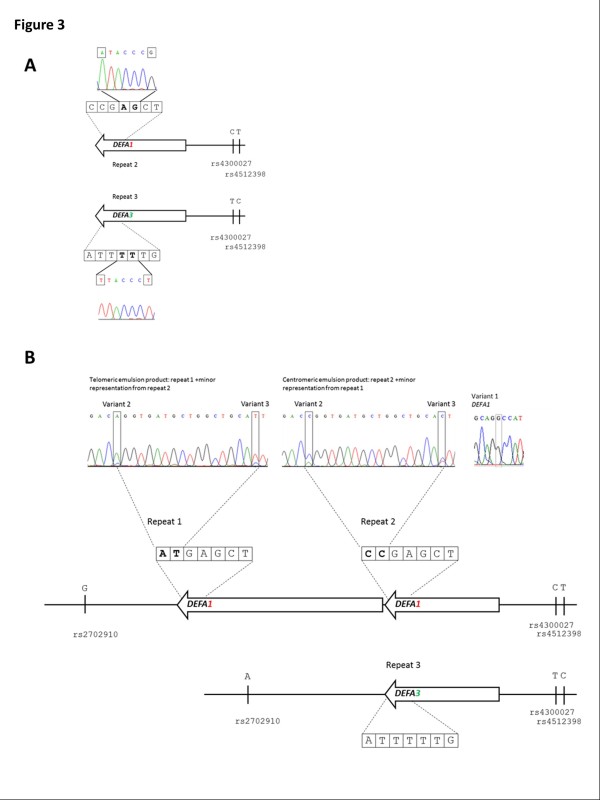
**Centromeric haplotype for NA06990.** Panel **A** shows the haplotypes for the most centromeric repeats for NA06990. Constituent haplotypes are resolved using allele-specific primers, specific to either the T or C at rs4512398. The phase of sequence variants within the gene (numbers 2 to 8; see additional Table 1) are shown boxed, from variant number 2 on the left to number 8 on the right. Variant positions 5 and 6 are shown in bold. The corresponding base in the sequence trace is shown boxed. Examination of the sequence at variant position 1 (data not shown) illustrated that T at rs4512398 is on the same chromosome as a copy of *DEFA1* and C at rs4512398 is in *cis* with a copy of *DEFA3*. Panel **B** illustrates some of the sequence information used to reconstruct the full haplotypes in this individual. For the centromeric emulsion product, allele-specific sequence using primer T at rs4512398 shows mixed positions at variant positions 2 and 3. At variant position 2, C is observed as the major peak, and A the minor peak and at variant position 3, C is the major peak with T as the minor peak. Sequencing of the entire fused product showed a C at variant position 1, which defines a copy of *DEFA1* thereby excluding inappropriate fusion with Repeat 3 on the other haplotype, which carries a copy of *DEFA3*. Sequencing from the telomeric direction (telomeric emulsion product) showed precisely reciprocal mixed positions (with A at variant 2 being the major product and C the minor). This is consistent with a major contribution from Repeat 1 and a minor contribution from Repeat 2. This reciprocity allowed reconstruction of this 2-copy haplotype. *DEFA1A3* repeat units are illustrated as arrows.

In order to build up a haplotype across the CNV, the nature of the end most repeats were defined first. Haplotypes for the centromeric end of the CNV region were established using the centromeric emulsion system (Figure 2B). This system determines the phase of flanking SNPs rs4300027 and rs4512398 with the gene in the centromeric (partial) repeat location. The condensed haplotypes were generated and the phase of flanking variants and nature of the gene in this position established by both non-discriminatory and allele-specific PCR and sequencing. Sequencing of the fused product with a non-discriminatory primer (CenR2N), a reverse primer within the single-copy flanking amplicon, which amplifies both condensed chromosomes, revealed eight heterozygous positions within the *DEFA1A3* portion of the fused product (including variant 1, a G/T single nucleotide variant that distinguishes *DEFA1* from *DEFA3*). This demonstrated that the centromeric partial repeats consist of one copy of *DEFA1* and one copy of *DEFA3* (denoted repeat 2 and repeat 3 respectively in Figures 2C and 3). Allele-specific PCR and sequencing was then used to establish the centromeric haplotypes as illustrated in Figure 3A.

Interestingly, as illustrated in Figure 3B, two of the eight variant positions (variant numbers 2 and 3) remained mixed after allele-specific PCR and sequencing with primer T at rs4512398, albeit with the secondary peak appearing at lower representation (Figure 3B: centromeric emulsion product). Sequencing of the entire allele-specific fused product showed a G at variant position 1, illustrating that the product was due to the fusion of the right-hand amplicon (containing centromeric flanking SNPs) to an additional copy of *DEFA1* and not *DEFA3,* and therefore not due to anomalous fusions appearing within the emulsion. F1 and R1 primers for the centromeric emulsion system (and also F2 and R2 of the telomeric system) are designed to amplify a copy-variable gene. The distance from the flanking SNPs to the next copy of *DEFA1* in repeat position 1 (chr8:6841698–6844134) (Figure 3) is 26kb. Given the physical distances involved and the expected average size of DNA in solution, this prompted investigation of whether the minor product represented fusion of the centromeric flanking amplicon to the copy of *DEFA1* in the next repeat (repeat 1). There was no mixed sequence for the other 6 heterozygous positions in this sequence and no mixed positions observed in the allele-specific sequence with primer C at rs4512398.

The genes at the telomeric end of the CNV were determined using the telomeric emulsion system. Sequence analysis showed that G at rs2702910 is on the same chromosome as a copy of *DEFA1* and A at rs2702910 is on the same chromosome as a copy of *DEFA3*. Examination of the allele-specific sequencing of the telomeric emulsion fused product using primer G at rs2702910 showed reciprocal mixed positions to the ones seen from the centromeric direction (Figure 3B: telomeric emulsion product). This provided confirmatory evidence that the sequence observed at lower representation on the trace is that of a second copy of the gene on the same chromosome.

Integration of sequence information from both the centromeric and telomeric emulsion systems allows the mapping of individual genes and associated repeat-specific sequence variants, and the phasing of these with flanking SNPs. Using emulsion haplotype fusion PCR, haplotypes could be reconstructed across the 38kb and 19kb of the 2-copy and 1-copy repeat unit respectively, as shown in Figure 3B. For the emulsion systems where primers are designed to amplify all copies of *DEFA1A3* present in an individual, we need to be sure that the major product is the result of the fusion of the flanking SNPs to the gene in the repeat unit next to the flanking SNPs. The success of the emulsion fusion PCR method has a close relationship with the average fragment size of the genomic DNA, because it depends on the flanking DNA and at least the first repeat unit being present on the same DNA molecule. However, because of randomly-positioned double-stranded DNA breaks, there is a decreasing probability that the second and subsequent repeats will also be on the same DNA molecule. Fusion from the second and third most repeats would therefore be predicted to happen more infrequently than fusion to the first, and hence these are observed as minor products. This observation can be further supported using samples such as NA06990 where the nature of the centromeric genes can be verified by long PCR or segregation.

The approach of integrating allele-specific sequence data from both telomeric and centromeric emulsion systems to build up a structural haplotype at *DEFA1A3* was then investigated in NA12760 and NA12878, two 5-copy individuals of unknown haplotype composition. Whilst the diploid copy number of these HapMap individuals had been established by PRT, no information on the haplotype copy number or haplotype composition was available. Information already determined concerning the ratio of number of copies of *DEFA1* to *DEFA3* was not used to inform inferences, but was subsequently checked for consistency.

**Figure 4:**
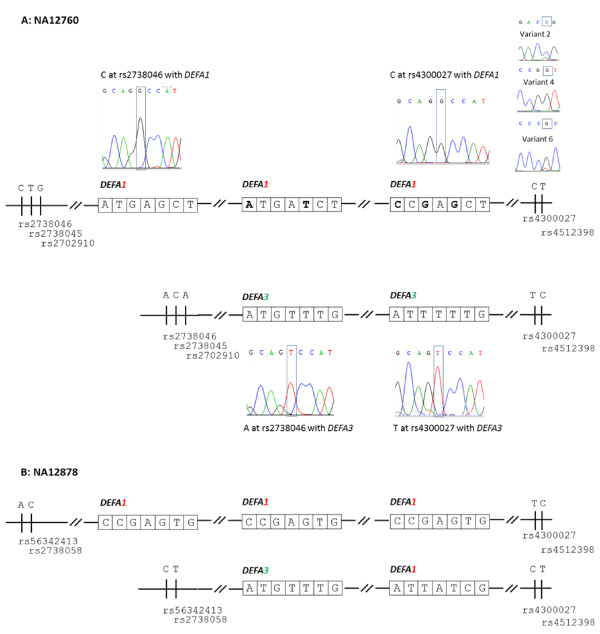
**The haplotypes defined by emulsion-fusion PCR at the *DEFA1A3 *locus on 8p23.1 are shown for NA12760 (panel A) and NA12878 (panel B).** Haplotype copy number and the phase of SNPs within the genes across this CNV were established by combining sequence information from the telomeric and centromeric emulsion systems that link SNPs flanking the CNV to the copy-variable genes in the repeat array. Panel A illustrates some of the sequence data used to define the structural haplotype. Sequence of variant 1, which distinguishes *DEFA1* (G) and *DEFA3* (T), are shown for the four end repeats for NA12670, for example C at rs2738046 is on the same chromosome as a copy of *DEFA1* and A at rs2738046 is on the same chromosome as a copy of *DEFA3*. Mixed positions (variants 2 and 6) and non-mixed positions (variant 4) are used provide spatial information regarding the five copies present in these individuals. The identity and position of the gene and sequence variants within each copy are shown. The phase of gene-specific sequence variants (numbers 2 to 8; see additional Table 1) are shown boxed, from variant number 2 on the left to number 8 on the right. Panel B shows the final structural haplotype for NA12878.

Sequence variants within the portion of *DEFA1A3* to be fused to flanking SNPs were identified in NA12760 and NA12878 by PCR and sequencing. In order to build up the structural haplotype for these 5-copy individuals, the nature of the most telomeric and centromeric repeats was established by emulsion PCR and allele-specific sequencing from both centromeric and telomeric emulsion systems respectively. Figure 4A shows portions of the sequence trace corresponding to the most telomeric and centromeric genes in the CNV using NA12760 as an example. Having established, in a sample of known haplotype composition, that mixed positions in the allele-specific sequence data were due to fusion of the flanking amplicon to the gene in the next repeat on the same chromosome, the sequence data for both 5-copy individuals was examined at the 8 variant positions within the gene. This is illustrated with variants 2 and 6 for the centromeric haplotype (see Figure 4A). Positions such as variant 4, which differs between the 2 haplotypes, but only shows a G in the allele-specific sequencing allows us to infer that a different variant of lower representation, when observed, is from the next gene on the same chromosome and not from the other haplotype. Combining allele-specific sequence data from both emulsion systems linking flanking regions of the CNV to sequence within the CNV demonstrated that both NA12760 and NA12878 are composed of a 2-copy and a 3-copy haplotype as illustrated in Figure 4A and 4B respectively, and has provided spatial information regarding the location of each copy of the gene and its associated variants. The results obtained were supported by the diploid *DEFA1*:*DEFA3* ratio. For a 3-copy allele consisting of two copy-variable 19kb full repeats and one 10kb centromeric partial repeat, this approach reconstructs a 59kb haplotype across the copy-variable locus. Whilst diploid copy number was known, no prior knowledge regarding haplotype copy number or composition was required, with data on flanking regions and the detection of phase obtained in a simple and straightforward manner.

## Conclusions

The emulsion fusion procedure described here has successfully fused two PCR products up to 1kb in length to determine the haplotype of variants and to reconstruct linear haplotypes across approximately 59kb at a copy-variable locus. This work has demonstrated a novel use for emulsion haplotype fusion PCR to establish structural haplotypes at complex genomic regions.

Using emulsion haplotype fusion PCR we have defined the haplotypes for the copy-variable *DEFA1A3* locus in three HapMap individuals, NA06990, NA12760 and NA12878. The diploid sequence of NA12878 has been extensively analysed as part of the 1000 genome project [45], and most recently whole genome fosmid data has been generated to resolve the diploid sequence information into phase haplotypes [23]. Yet despite this “gold standard” approach to genome-wide definition of haplotypes, it has not yet allowed simple reconstruction of haplotypes at multiallelic copy-variable regions, such as the *DEFA1A3* locus (M. Hoehe, personal communication). Copy variable regions are often studied from a diploid copy number perspective with positional information and sequence variants within each copy repeat neglected, primarily due to the difficulty in resolving sequence relationships for these regions. These regions are often poorly understood especially in terms of dissecting diploid copy number states into haploid allelic contributions, yet the position of each of these genes and their local sequence variants within the array may have an effect on expression [46]. Determination of the combinations of sequence variants within copy-variable repeat units can, in parallel, provide valuable information on the evolutionary history of these regions by clarifying true structural relationships between alleles.

Previous use of haplotype emulsion fusion PCR emphasised its use in genotyping two or three specific SNPs across a distance greater than amenable to long PCR [34,36]. In this study, emulsion haplotype fusion PCR was used to build a structural haplotype across a complex and structurally variable region using the *DEFA1A3* locus as an example. Amplicons telomeric and centromeric to the CNV were designed to fuse to an amplicon within the CNV thus providing haplotypes at each end of the CNV. Integration of sequence information was then used to reconstruct the haplotype. For many DNA samples studied in the laboratory, information on diploid copy number of a copy-variable gene can be straightforwardly ascertained. In addition, if family members are available haplotype copy number can be established through analysis of segregation. What remains to be established is positional information relating to the genes and any associated variants. To answer this, allele-specific sequence data from two emulsion fusion PCR systems, one telomeric to the CNV and the other centromeric to the CNV were used to define which gene and associated variants resided in the end repeat positions. Mixed positions in the allele-specific product provided evidence of fusion of the flanking amplicon to the amplicon from the gene of the second repeat on the same chromosome. Our experience with samples of known haplotype suggests that products detected as minor peaks on haplotype-specific sequence traces can indicate the sequence of the second repeat unit adjacent to the flanking product. In the case of the two-copy haplotypes observed in this study, data from the centromeric emulsion can complement that from the telomeric emulsion- i.e., from the centromeric direction, the fusion of the gene (and variants) in the centromeric partial repeat is the major product with the next gene on the same chromosome (a further 19kb away) being the minor product, and vice versa from the telomeric direction. This work led to the construction of a structural haplotype of 59kb across the CNV, in which the relative positions of different gene sequence variants are defined. In the case of NA12878, where the 3-copy haplotype contained identical copies of *DEFA1,* haplotype structure was supported by total diploid CN, ratio of *DEFA1*:*DEFA3* and using information obtained for the 2-copy haplotype.

The ability to fuse amplicons of 1kb and longer means that more SNPs can be phased simultaneously. Not only is this particularly important if not all variants within the copy-variable region are informative for all samples or all repeat units, but the longer amplicon derived from the single-copy flanking region can also be designed to include multiple SNPs that can act as internal controls for checking allele-specificity of the PCR. For any locus, emulsion systems can be easily and straightforwardly designed with no restriction on primer position, to produce sequence data that is straightforward to interpret. Whilst this study has established a structural haplotype for a chromosome with 3 copies of *DEFA1A3*, this procedure is not necessarily limited to low copy samples. If required, repeat-specific variants can be exploited in an emulsion system to “walk” between repeats across a higher-copy haplotype.

Emulsion haplotype-fusion is a relatively simple and versatile procedure for molecular haplotyping, but has had a limited uptake in practice. Previous protocols have used a variety of downstream methods to determine the haplotype. For example, Turner *et al.* used a bridging oligonucleotide, adjacent to the two SNPs under interrogation, to detect the haplotype in the fused product made in the emulsion PCR stage. Specific oligos of different length were then used to target each possible base of the SNP and ligated to the common bridging oligo. After amplification with universal primers, each haplotype had a characteristic size and as such was determined by capillary electrophoresis. Whilst successful and applicable to high-throughput haplotype determination, the scheme imposes limits on the position of the primers and the number of SNPs that can be examined. The constraints imposed by the post-fusion detection of the haplotype in previously published approaches, for example on the position of primers and bridging oligonucleotides, the use of specialist equipment (e.g. fluorescence microscope for bead haplotyping) or modified oligonucleotides [34-36] has in turn meant that only small amplicons have been amplified and subsequently condensed into a haplotype, and that information from very few SNPs has been captured [34,36]. These post-fusion detection methods may have been adopted because previous methods were not capable of reliably generating fused product of sufficient purity to analyse by direct sequencing, and if so this limitation seems to have been removed by our adoption of nested primers in the secondary PCR. Building on the original principle of fusion PCR, the procedure developed here is characterised by nesting of the secondary product and a simple post-fusion detection of phase by direct sequencing. Our approach with larger amplicons fused in an emulsion allows a greater number of SNPs to be examined simultaneously and more flexibly in a single reaction with no restriction on primer position. Creating fused products from elements up to about 50kb apart by these methods would not require special treatment of genomic DNA, and so could be easily applied to the kinds of genomic DNA preparation generally held in banks of clinically-derived samples.

The development of next-generation sequencing technologies has allowed the sequencing of individual genomes to become a reality, documenting the true extent of variation contained within them both with respect to sequence and structural variation. Despite this progress, there remains the need to establish phase of variants, including the haplotypes at CNVs, in order to provide a complete assessment of any individual’s genome. Without approaches to reconstruct spatial information in human genome sequences, our picture of the genome will remain collapsed and incomplete.

## Competing interest

The authors declare no competing interests.

## Authors’ contributions

JT and JA designed the study; JT did the experimental work and data analysis; both authors contributed to the final manuscript.

## Supplementary Material

Additional file 1 Includes additional text and tables. Click here for file

Additional file 2 **Additional figures.** Contains additional figures 1, 2 and 3. Click here for file
