# Supplementary material for: Determination of haplotypes at structurally complex regions using emulsion haplotype fusion PCR
Source: BMC Genomics. 2012 Dec 11;13:693. doi: 10.1186/1471-2164-13-693 (PMC3543183; doi:10.1186/1471-2164-13-693)
Supplement: Additional file 1 — Includes additional text and tables. [file 1471-2164-13-693-S1.docx]

**Additional file 1**

**Background**

Prior to use of the emulsion haplotype fusion PCR approach used for reconstructing haplotypes at *DEFA1A3*, initial work centred on the *CCL3L1*/*CCL4L1* locus as a test system.

*CCL3L1*/*CCL4L1* locus

The region consists of an approximately 90kb repeat unit containing two genes, *CCL3L1* and *CCL4L1*. *CCL3L1* and *CCL4L1* show 95% nucleotide sequence identity to their respective paralogues *CCL3* and *CCL4*, which do not vary in copy number [[1](#_ENREF_1)]. Each copy of the repeat unit is flanked by copies of an unrelated gene *TBC1D3*. The structure of the region according to the UCSC genome browser (2006 assembly)[[2](#_ENREF_2)] is shown in Supplementary figure 1A. In Europeans, the copy number of this region commonly ranges from 0 to 4 [[3](#_ENREF_3)]. Approximately 2% of the UK population have two null alleles of *CCL3L1* and *CCL4L1* and therefore lack *CCL3L1* and *CCL4L1* entirely. These are denoted as zero-copy samples and provide an important control for specificity of the primers.

*CCL3L1*/*CCL4L1* copy number and sequence analysis.

Prior to this work, HapMap CEU samples had been extensively studied from a copy number and sequence point of view. The copy number of this locus was measured by Paralogue ratio test (PRT) [[3](#_ENREF_3)]. In addition, *CCL3L1* and *CCL4L1* were individually amplified and sequenced with single nucleotide variants identified and validated (JALA and Somwang Janyakhantikul, unpublished data). Individuals that possessed 1 or 2 copies of *CCL3L1* and *CCL4L1*, in which the individual gene haplotype was unambiguously established, were identified.

Segregation analysis and allele-specific sequencing.

HapMap CEU trio from family 1254, which comprises father (NA12812), child (NA12801) and mother (NA12813) whose diploid copy numbers (CN) for *CCL3L1*/*CCL4L1* are 2, 1 and 1 respectively were chosen to study further. *CCL3L1/CCL4L1* copy number inheritance transmission patterns were inferred by segregation of microsatellites within the copy variable region in conjunction with genetic data from the CEPH linkage database (http://www.cephb.fr/en/cephdb/). This allowed the determination of the *CCL3L1/CCL4L1* copy number per chromosome. By means of segregation analysis and allele-specific sequencing, sequence variants could be assigned to haplotypes within *CCL3L1* and separately within *CCL4L1*. In this HapMap trio, segregation analysis could also determine the combination of the *CCL3L1* and *CCL4L1* haplotypes into a complete haplotype for the two genes within the same repeat unit. Both child (NA12801) and mother (NA12813) have a diploid CN of 1, and therefore both possess a chromosome with a single repeat allele (1-copy chromosome) and chromosome lacking the *CCL3L1*/*CCL4L1* repeat unit i.e. a zero-copy chromosome or null allele. Segregation analysis demonstrated the transmission of the zero-copy chromosome from mother to child, implying that the 1-copy haplotype possessed by the child has been transmitted from the father. This provides evidence for the father having 1 copy of the repeat unit per chromosome, as opposed to possessing a zero-copy chromosome and a 2-copy chromosome. Sequencing in the child allows the identification of which one-copy haplotype is transmitted from the father, and hence the haplotype content of the second untransmitted paternal copy can be deduced. Therefore through sequencing in all three individuals, the haplotype of sequence variants within *CCL3L1* and *CCL4L1* for each copy is known. Given this knowledge, these samples provided a vital resource to test the accuracy of the emulsion haplotype fusion PCR approach outlined below in linking sequence and maintaining phase.

Primer design

Primers were designed to amplify *CCL3L1* and *CCL4L1* but discriminate against their respective paralogues (*CCL3*/*CCL4*). A further complication arises from the presence of a 5’ truncated pseudogene of *CCL3L1* in some copy variable repeat units [[1](#_ENREF_1)]. Since sequences common to full length *CCL3L1* and the pseudogene cannot be distinguished, primers were designed so that at least one primer, either F1 or R1, was positioned in sequence not shared between full length *CCL3L1* and the truncated version. A zero-copy sample was included in all PCR experiments as a control for primer specificity to demonstrate specific amplification from *CCL3L1* and *CCL4L1* and not their respective paralogues, *CCL3* and *CCL4*.All primers are shown in supplementary table 2.

Primer concentration

In order to establish an optimum concentration for F2’R1, experiments were carried out with a range of F2’R1 concentrations from 10nM to 250nM, in both non-emulsion and emulsion PCR.

**Results and discussion**

Emulsion haplotype fusion PCR for the 2025bp *CCL3L1*/*CCL4L1* test system (illustrated in supplementary figure 1) was carried out for NA12812 and controls. Sequencing of the nested PCR product from NA12812 was then carried out to resolve the phase of variants in the fused amplicon. This was investigated using a combination of both non-discriminatory and allele-specific PCR primers designed to sequence the product in its entirety and to make sure the fusion breakpoint was covered. Due to the length of the fused product (1740bp), sequencing to determine phase was established in three sections. Sequencing with an allele-specific reverse primer (ASPCR2086R) located within the *CCL4L1* portion of the fused product provides sequence information across the fusion junction and hence provides phase information coupling SNPs in *CCL3L1* to those in the *cis*-adjacent copy of *CCL4L1* (see supplementary figure 1A for position of primer). A portion of this sequence is shown in Supplementary figure 1C. Sequencing with a non-discriminatory primer 2086R reveals a mixed position at rs1804185 in this 2-copy individual (Supplementary figure 1C: panel i). Sequencing with allele-specific 2086R-T demonstrates that T at position rs2277660 in *CCL4L1* is on the same haplotype as C at rs1804185 in *CCL3L1* (Supplementary figure 1C: panel ii). Sequencing with ASPCR2086R-G demonstrates that G at position rs2277660 in *CCL4L1* is on the same haplotype as T at rs1804185 in *CCL3L1* (supplementary Figure 1C: panel iii). Since the complete haplotype of *CCL3L1* and *CCL4L1* for each 1-copy chromosome possessed by NA12812 was derived through segregation and sequencing analysis prior to this work (see Supplementary Information), this could be used to support data obtained from the emulsion experiment. The two 1-copy haplotypes for NA12812 are shown in supplementary figure 1D. Sequencing across both the fusion junction and within the *CCL3L1* and *CCL4L1* portions verifies that the emulsion fusion procedure has not created recombinant PCR products in which the haplotypes have been scrambled.

Clarification of phase of SNPs within *CCL3L1* and within *CCL4L1*.

The phase of SNPs within the *CCL3L1* portion of the fused product was verified using PCR and sequencing with either F1N or allele-specific primers ASPCR532F-A or ASPCR532F-G with R2N as the reverse primer. Sequencing of the fused product with F1N reveals mixed positions at rs17850251 (supplementary figure 2: panel IA) and rs1804185 (supplementary figure 2: panel IB). When the same first round product is amplified and subsequently sequenced using either allele-specific primer ASPCR532F-A or ASPCR532F-G in the second round, the variant bases which are on the same haplotype as the discriminatory base at the 3’ position of the allele-specific primer are revealed (supplementary figure 2: panel IIA and B; panel IIIA and B). The haplotype revealed by emulsion fusion PCR is supported by previous *CCL3L1* allele-specific sequence data derived from genomic DNA and confirms that the phase of the sequence variants within *CCL3L1* is maintained in the fused PCR product.

The haplotype of sequence variants within *CCL4L1* were similarly established by sequencing of the F1N to R2N (1740bp) fusion PCR product with either R2N or allele-specific primer ASPCR1467R-T or ASPCR1467R-G. Sequencing with R2N reveals mixed positions at chromosome 17:31664359 [[2](#_ENREF_2)] (March 2006 assembly) and rs2277660 in this 2-copy individual (supplementary figure 3: panel IA and 1B). Haplotypes were revealed by PCR and sequencing with F1N and either allele-specific primer ASPCR1467R-T or ASPCR1467R-G as the reverse primer (supplementary figure 3: panel IIA and B; panel IIIA and B). As is the case for the *CCL3L1* portion of the fused product, the use of previous data derived from genomic DNA verifies that the phase of SNPs within this part of the fused product is maintained in the emulsion fusion PCR.

No evidence of alternative haplotypes, in which the known phase was not maintained, was observed, nor were there any mixed positions on allele-specific sequence traces to indicate incomplete resolution or illegitimate junctions.

In order to successfully produce 1kb amplicons and longer in an emulsion the design and inclusion of an additional primer (F2A, designed at locus 2) in the emulsion PCR stage was necessary. Previous emulsion haplotype fusion methods [[4](#_ENREF_4), [5](#_ENREF_5)] have used three primers in the emulsion stage of the PCR, allowing exponential amplification to occur between F1 and F2’R1 (locus 1, or in this test case *CCL3L1*) and a single-stranded product to be synthesised from the R2 primer (locus 2, or in this test case *CCL4L1*). The F2A primer was designed to enable exponential amplification between F2A and R2 and thus remove the reliance on linear synthesis from the R2 primer. The inclusion of F2A was not necessary to produce satisfactory yields for amplicons of less than 1kb.

Designing an additional primer, designated F2A, which is used in the emulsion PCR stage means that a longer fused amplicon can be reliably produced. The addition of F2A, set back from the “F2” region, means that early in the process a double-stranded product is exponentially amplified from the right-hand amplicon or locus 2, and thus removes the reliance on the generation of a linear product from R2. We designed the primer F2A set back from the F2 sequence, rather than using the F2 sequence itself, to avoid two complementary sequences (F2’R1 and F2) interfering with each other directly in the emulsion PCR. We assume that inclusion of an F2A primer improves the efficiency and specificity of the fusion stage by promoting exponential, rather than linear, amplification of the R2 amplicon. The design and addition of F2A is not necessary for shorter condensed products; nevertheless, even when fusion could be successfully achieved without it, the yield of fused product was greatly enhanced by F2A - for the shorter-product systems carried out as a proof of principle, addition of this primer meant that the fused product appeared after a total of 45 PCR cycles as opposed to 65 cycles without F2A (data not shown).

1. Modi WS: **CCL3L1 and CCL4L1 chemokine genes are located in a segmental duplication at chromosome 17q12**. *Genomics* 2004, **83**(4):735-738.

2. Kent WJ, Sugnet CW, Furey TS, Roskin KM, Pringle TH, Zahler AM, Haussler D: **The human genome browser at UCSC**. *Genome Res* 2002, **12**:996 - 1006.

3. Walker S, Janyakhantikul S, Armour JAL: **Multiplex Paralogue Ratio Tests for accurate measurement of multiallelic CNVs**. *Genomics* 2009, **93**(1):98-103.

4. Turner DJ, Hurles ME: **High-throughput haplotype determination over long distances by haplotype fusion PCR and ligation haplotyping**. *Nat Protocols* 2009, **4**(12):1771-1783.

5. Wetmur JG, Kumar M, Zhang L, Palomeque C, Wallenstein S, Chen J: **Molecular haplotyping by linking emulsion PCR: analysis of paraoxonase 1 haplotypes and phenotypes**. *Nucleic Acids Res* 2005, **33**(8):2615-2619.

**Figure legends for additional figures**

**Additional figure 1.**

Schematic diagram of the *CCL3L1*/*CCL4L1* locus PCR products, sequence variants, primers and haplotypes to illustrate the emulsion haplotype fusion procedure. Panel A shows the position, physical distances and sizes of the genes *CCL3L1* and *CCL4L1*. *CCL3L1* and *CCL4L1* positions are denoted as chromosome 17:31647956-31649843 and chromosome 17:31664147-31665959 respectively on the UCSC Human Genome browser, March 2006 assembly [[2](#_ENREF_2)]. The arrangement of primers used in the first round of emulsion haplotype fusion PCR and informative sequence variants present in the two-copy individual NA12812 are also illustrated. Whilst local phase of variants within each 1kb amplicon can be determined by allele-specific PCR, the phase of variants in *CCL3L1* with those in *CCL4L1*, 16kb away is unknown. The agarose gel in panel B illustrates the 1740bp fused PCR product amplified using nested primers F1N and R2N in the second round of PCR (see supplementary table 2). HapMap CEU samples are denoted with the prefix NA. Lane 2 is control DNA ECACC C0034. NA10846 is a *CCL3L1*/*CCL4L1* zero-copy individual. 1^o^ PCR blank comprises an emulsion PCR mix with no DNA in the first round of PCR amplified in the first round of PCR, extracted and subsequently re-amplified in the second round of PCR. NCE is a non-cycle emulsion control (see Haplotype fusion PCR section of the materials and methods for details) and 2^o^ PCR blank is a no-DNA control for the second stage of PCR. Panel C shows non-discriminatory and allele-specific sequencing of the fused PCR product. Sequencing with a non-discriminatory primer 2086R (panel i) reveals a heterozygous position at rs1804185 in this 2-copy individual. Sequencing with ASPCR2086R-T demonstrates that T at position rs2277660 in *CCL4L1* is on the same haplotype as C at rs1804185 in *CCL3L1* (panel ii). Sequencing with ASPCR2086R-G demonstrates that G at position rs2277660 in *CCL4L1* is on the same haplotype as T at rs1804185 in *CCL3L1* (panel iii). Variant bases are shown boxed. The two haplotypes for NA12812 are shown in panel D.

**Additional figure 2.** Portion of *CCL3L1* sequence of fused PCR product from NA12812 (panel I). This individual possesses 2 copies of *CCL3L1* and *CCL4L1*. Sequencing of the secondary product with F1N reveals heterozygous positions at rs17850251 (panel I-A) and rs1804185 (panel I-B). Variant bases are highlighted by a circle. When the same first round product is amplified using allele-specific primers ASPCR532F-A or ASPCR532F-G in the second round, the 2-copies are split into component haplotypes (sections II and III). These data indicate that base A at rs2944 is on the same haplotype as A at rs17850251 and T at rs1804185 (panels II-A and II-B respectively) and that base G at rs2944 is on the same haplotype as G at rs17850251 and C at rs1804185 (panels III-A and III-B respectively).

**Additional figure 3.**

Portion of *CCL4L1* sequence of fused PCR product from NA12812 (panel I). Sequencing with R2N reveals heterozygous positions at chromosome 17:31664359 (Human Genome browser [[2](#_ENREF_2)], March 2006 assembly) and rs2277660 in this 2-copy individual (panel IA and IB respectively). Haplotypes are revealed by sequencing with allele-specific primer ASPCR1467R-T or ASPCR1467R -G which associate T at position rs3744597 with A at chromosome 17:31664359 (panel IIA) and G at rs2277660 (panel IIB) and G at position rs3744597 with C at chromosome 17:31664359 (panel IIIA) and T at rs2277660 (panel IIIB). Variant bases are highlighted by a circle. The sequence variant at position 17:31664359 is not present in dbSNP129.

**Additional table 1.**

Sequence variants present in *DEFA1A3* identified by PCR and sequencing. Where no entry in dbSNP 135 was available, browser coordinates for the three copies of *DEFA1A3* are provided (UCSC Feb. 2009 version).

**Additional table 2.**

Primers used in the first and second round of emulsion haplotype fusion PCR. For CCL3L1/CCL4L1, primers used in the emulsion are shown in italics. Nested primers (denoted as F1N and R2N), allele-specific PCR primers (denoted with an *) and respective annealing temperatures used in the second round of amplification are shown. Reverse primers are shown in bold.

Additional table 1

| Variant number | Variant  Bases | dbSNP build 135 |  |
| --- | --- | --- | --- |
| 1 | G/T | rs145076681 | Variant base that distinguishes *DEFA1* from *DEFA3* |
| 2 | A/C | rs148298380 |  |
| 3 | T/C | rs139164630 |  |
| 4 | G/T | rs147777993 |  |
| 5 | A/T | rs77140339 |  |
| 6 | T/G | rs77649030 |  |
| 7 | C/T | chr8:6835780; 6845897; 6873996 |  |
| 8 | G/T | rs77855524 |  |

Additional table 2

| Locus | Emulsion fusion system | Primer name | Primer sequence (5’-3’) | Annealing temperature used in 2^0^ PCR |
| --- | --- | --- | --- | --- |
| *CCL3L1* |  | *F1* | *CACTCGGTTGTCACCAGACACAC* |  |
|  |  | *F2’R1* | *TGTCATGGCTCCTGAAGCTAGCTGCCTGCCCTCCTCAACCACTCA* |  |
|  |  | *R2* | *CCCAAAACAGGCCCCCTTTA* |  |
|  |  | *F2A* | *CGCCTGCACCTCCCTCATCTTT* |  |
|  |  | F1N | GTGGCTGTTTGGCAATAACC | 68^o^C |
|  |  | **R2N** | GCGAGGAAGCTTCCTCGCG | 68^o^C |
|  |  | **ASPCR2086G*** | AAGAGGTTTTCTCAGAGGTGAGG | 65^o^C |
|  |  | **ASPCR2086T*** | CAAAGAGGTTTTCTCAGAGGTGAGT | 65^o^C |
|  |  | **2086R** | GGTGGCAAAGAGGTTTTCTCAGA | 65^o^C |
| *DEFA1A3* | Centromeric 1 | F1N | GCTGGTATTCTGCAATAGCAG | 65^o^C |
|  |  | **CenR2N** | GCTGAAGGATGAGACCCTGT | 65^o^C |
|  |  | **rs4512398C*** | ATCAGGCCAGCTCATGAGG | 65^o^C |
|  |  | **rs4512398T*** | ATCAGGCCAGCTCATGAGA | 64^o^C |
|  | Telomeric 1 | F1N | CCAGTTGAGGACGATGGGAT | 65^o^C |
|  |  | **TelR2N** | GGTTCCAGAGTTGGGTCTCA | 65^o^C |
|  |  | rs2738046C* | CTCATCTGCCCCCTTCCC | 71^o^C |
|  |  | rs2738046A* | CTCATCTGCCCCCTTCCA | 71^o^C |
|  |  | rs2702910G* | GATTTTTATTGATTTTTAAAGCAATG | 68^o^C |
|  |  | rs2702910A* | GATTTTTATTGATTTTTAAAGCAATA | 68^o^C |
|  | Telomeric 2 | F1N | ACAGGAAGCCCATGTCTCTTCC | 65^o^C |
|  |  | **TelR2N** | GGTTCCAGAGTTGGGTCTCA | 65^o^C |
|  |  | rs56342413A* | GAAGCCCATGTCTCTTCCA | 72^o^C |
|  |  | rs56342413C* | GAAGCCCATGTCTCTTCCC | 72^o^C |

*allele-specific primers
