# Supplementary material for: Determination of haplotypes at structurally complex regions using emulsion haplotype fusion PCR
Source: BMC Genomics. 2012 Dec 11;13:693. doi: 10.1186/1471-2164-13-693 (PMC3543183; doi:10.1186/1471-2164-13-693)

Additional Figure 1

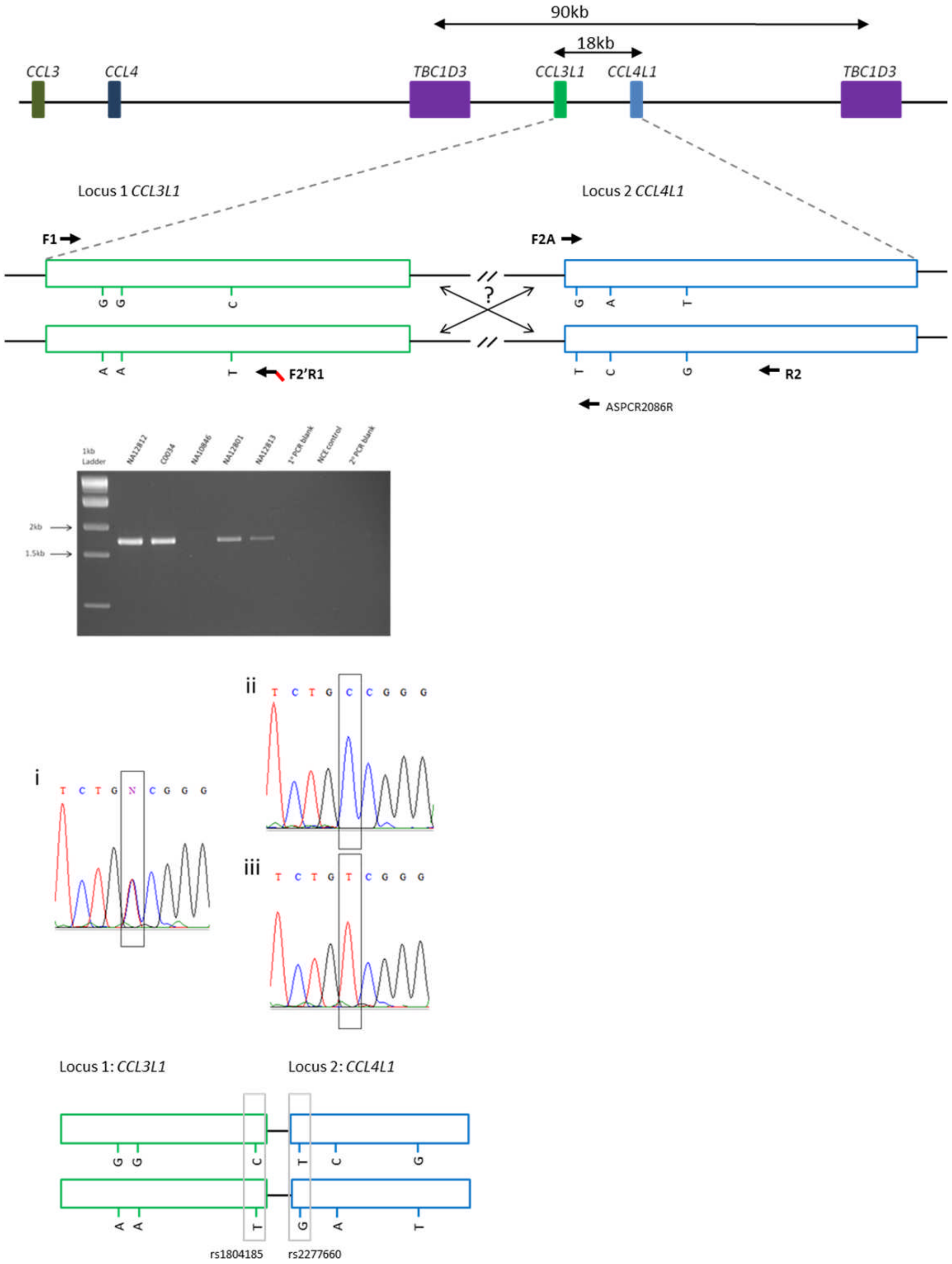

Additional Figure 2

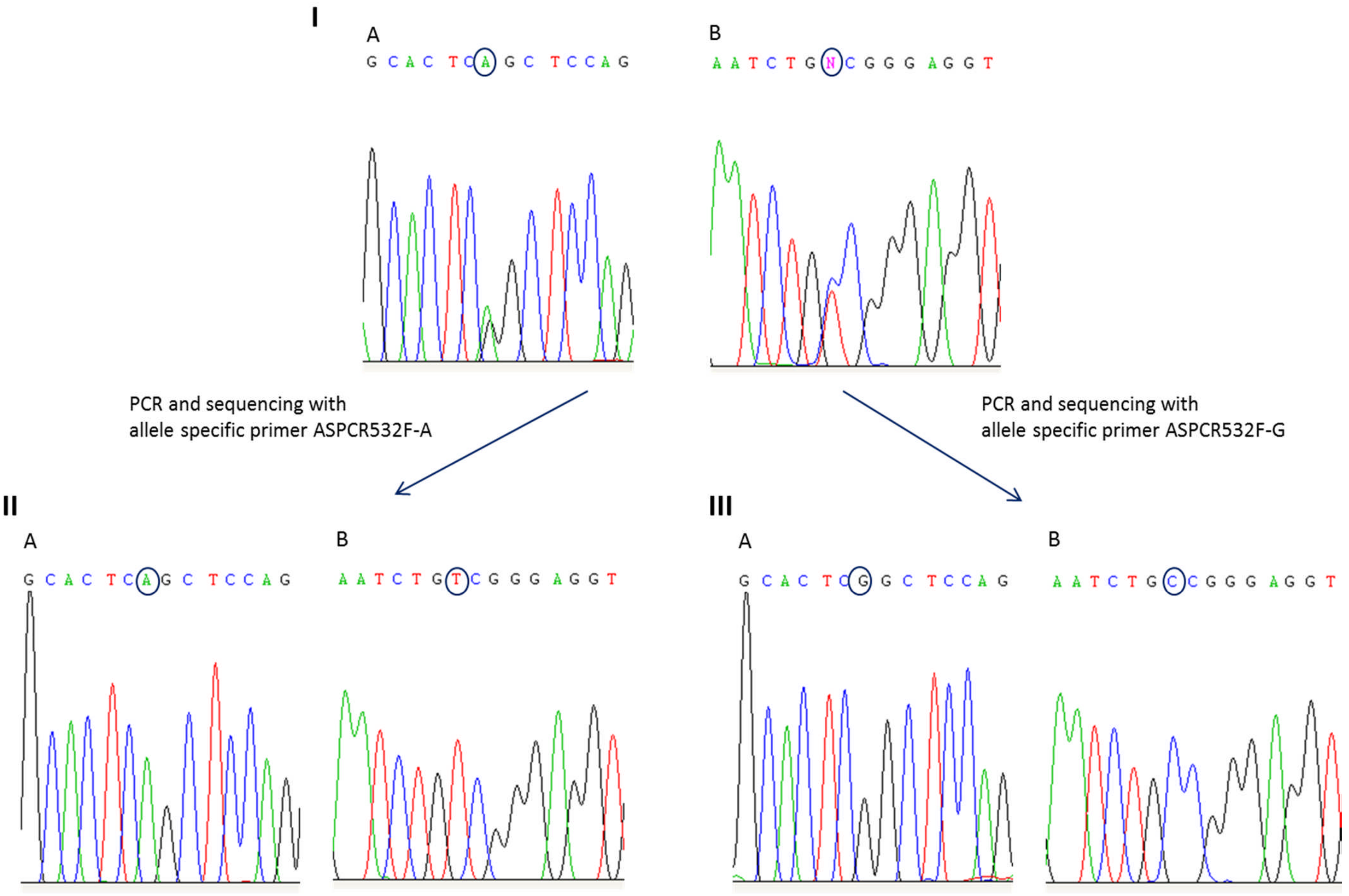

Additional Figure 3

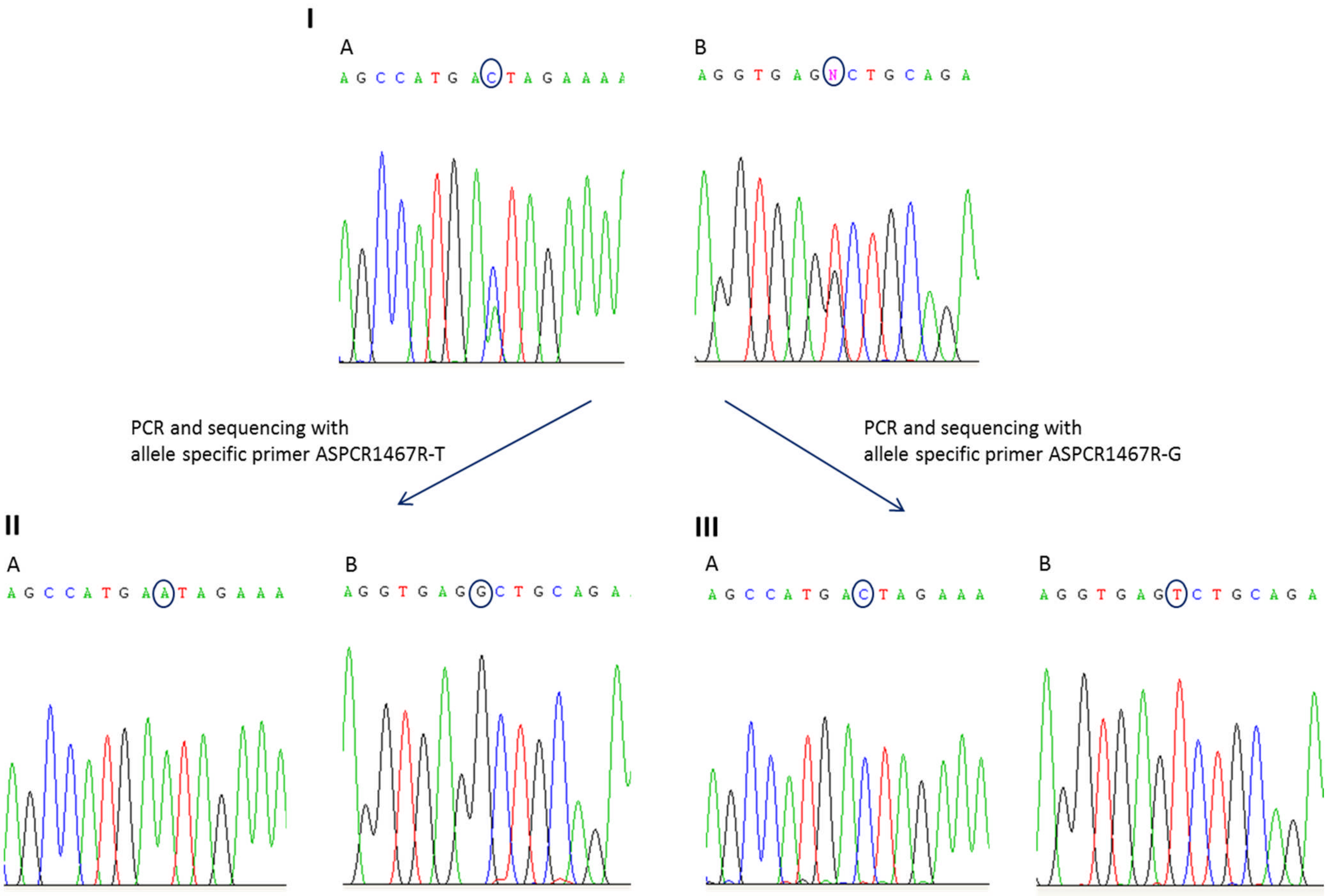

Supplement: Additional file 2 — Additional figures. Contains additional figures 1, 2 and 3. [file 1471-2164-13-693-S2.pdf]
